# MEK1 Inhibitor Combined with Irradiation Reduces Migration of Breast Cancer Cells Including miR-221 and ZEB1 EMT Marker Expression

**DOI:** 10.3390/cancers12123760

**Published:** 2020-12-14

**Authors:** Nataša Anastasov, Elisabeth Hirmer, Marbod Klenner, Jessica Ott, Natalie Falkenberg, Xuanwen Bao, Lisa Mutschelknaus, Simone Moertl, Stephanie Combs, Michael J. Atkinson, Thomas Schmid

**Affiliations:** 1 Institute of Radiation Biology, Helmholtz Zentrum München-German Research Center for Environmental Health, 85764 Neuherberg, Germany; elisabeth.hirmer@tum.de (E.H.); Marbod.Klenner@med.uni-muenchen.de (M.K.); jessica.ott@tum.de (J.O.); xuanwen.bao@tum.de (X.B.); lisa.mutschelknaus@gmail.com (L.M.); smoertl@bfs.de (S.M.); m.j.atkinson@tum.de (M.J.A.); 2 Institute of Biological and Medical Imaging, Helmholtz Zentrum München-German Research Center for Environmental Health, 85764 Neuherberg, Germany; 3 Institute of Radiation Medicine, Helmholtz Zentrum München-German Research Center for Environmental Health, 85764 Neuherberg, Germany; stephanie.combs@tum.de (S.C.); thomas.schmid@helmholtz-muenchen.de (T.S.); 4 Institute of Pathology, Technical University of Munich (TUM), 81675 Munich, Germany; natalie.falkenberg@gmx.de; 5 Clinical Research Unit, Department of Obstetrics and Gynecology, Technical University of Munich (TUM), 81675 Munich, Germany; 6 Federal Office of Radiation Protection, 85764 Oberschleissheim, Germany; 7 Department of Radiation Oncology, School of Medicine, Technical University of Munich (TUM), 81675 Munich, Germany; 8 Radiation Biology, Technical University of Munich, 81675 Munich, Germany

**Keywords:** MEK1 inhibitor (TAK-733), miR-221, ZEB1, migration assays, 3D-microtissue assays, breast cancer and radiation

## Abstract

**Simple Summary:**

Combined chemotherapy and radiotherapy are an effective treatment for invasive breast cancer. However, some studies suggest that such interventions may increase the risk of metastasis. Cell metastatic behavior is highly dependent on RAS-RAF-MEK pathway and its downstream target activation, including miR-221 overexpression and epithelial-to-mesenchymal transition (EMT). By using MEK1 inhibitor (TAK-733) in combination with radiation therapy for breast cancer cells, significant decrease in migration capacity, including reduction of miR-221 and EMT (ZEB1) marker expression was observed. miR-221 holds great potential as therapeutic biomarker and target for new drug developments, however more insight into efficiency of miR-221 inhibition needs to be followed in the future.

**Abstract:**

The miR-221 expression is dependent on the oncogenic RAS-RAF-MEK pathway activation and influences epithelial-to-mesenchymal transition (EMT). The Cancer Genome Atlas (TCGA) database analysis showed high gene significance for ZEB1 with EMT module analysis and miR-221 overexpression within the triple-negative breast cancer (TNBC) and HER2+ subgroups when compared to luminal A/B subgroups. EMT marker expression analysis after MEK1 (TAK-733) inhibitor treatment and irradiation was combined with miR-221 and ZEB1 expression analysis. The interaction of miR-221 overexpression with irradiation and its influence on migration, proliferation, colony formation and subsequent EMT target activation were investigated. The results revealed that MEK1 inhibitor treatment combined with irradiation could decrease the migratory potential of breast cancer cells including reduction of miR-221 and corresponding downstream ZEB1 (EMT) marker expression. The clonogenic survival assays revealed that miR-221 overexpressing SKBR3 cells were more radioresistant when compared to the control. Remarkably, the effect of miR-221 overexpression on migration in highly proliferative and highly HER2-positive SKBR3 cells remained constant even upon 8 Gy irradiation. Further, in naturally miR-221-overexpressing MDA-MB-231 cells, the proliferation and migration significantly decrease after miR-221 knockdown. This leads to the assumption that radiation alone is not reducing migration capacity of miR-221-overexpressing cells and that additional factors play an important role in this context. The miR-221/ZEB1 activity is efficiently targeted upon MEK1 inhibitor (TAK-733) treatment and when combined with irradiation treatment, significant reduction in migration of breast cancer cells was shown.

## 1. Introduction

Adjuvant radiation therapy for breast cancer (stage I–III) improves disease-free survival and overall survival [1]. Combination of chemotherapy with radiation treatment offers a cost-effective first-line treatment that prolongs 15-year survival for breast cancer patients [1]. Triple-negative breast cancer (TNBC) and human epidermal growth factor receptor-2 (HER2) positive tumours, both have a particularly poor prognosis, with higher rates of recurrence when compared to hormone ER (estrogen receptor) and PR (progesterone receptor) positive cases [2]. Recurrent tumours are often resistant to further radio- chemo- therapies (RCT) and may show increased migratory (metastatic) potential [3]. Consequently, there is no standard therapy for the breast cancer patients with recurrent or metastatic (advanced) breast cancer and it remains a great challenge to select an appropriate treatment [4,5].

In order to find a successful therapeutic approach for advanced breast cancers, the focus should be on the mechanisms involved in cell invasion and metastasis, including regulation of the epithelial-to-mesenchymal transition (EMT). One of the signaling pathways involved in tumor progression and EMT regulation is the RAS-RAF-MEK-MAPK pathway [6,7]. These kinases are overexpressed and hyperactive in various types of cancer. They regulate diverse cellular processes such as proliferation, migration, metastasis, resistance to chemotherapy, and EMT [8]. Furthermore, the concept that non-coding RNAs including miRNAs can promote transformation to more aggressive cancer phenotypes with poor prognosis has already been documented [9].

We have previously shown that the expression of miR-221 was associated with higher risk of metastasis in advanced breast cancers [10] and miR-221 was considered to be a prognostic marker for distinguishing subgroups particularly in advanced (lymph node-positive and HER2-positive) breast cancers [10]. Additionally, miR-221 overexpression may promote the EMT transition by targeting Notch3 [11] and via targeting the 3′UTR of GATA transcriptional repressor (TRPS1), leading to increased cell migration and invasion [12,13] in breast cancer cells. Moreover, it has been reported that miR-221 negatively regulates ER [14]. Increased resistance to tamoxifen, a drug successfully used to treat women with ER-positive breast cancer, was associated with ER and p27 inhibition upon miR-221 overexpression [15,16]. Furthermore, it has been shown that miR-221 confers radioresistance in glioblastoma cells [17]. We now report that miR-221 overexpression is associated with greater cellular migration capacity of breast cancer cells upon radiation treatment. As miR-221 acts downstream of the oncogenic RAS-RAF-MEK pathway, MEK1 inhibitor (TAK-733) was combined with irradiation treatment for subsequent miR-221 functional and phenotypic analysis including cell migration and proliferation activity.

## 2. Results

### 2.1. Differential miR-221 Expression in Breast Cancer Subgroups and Cell Lines

**Figure 1:**
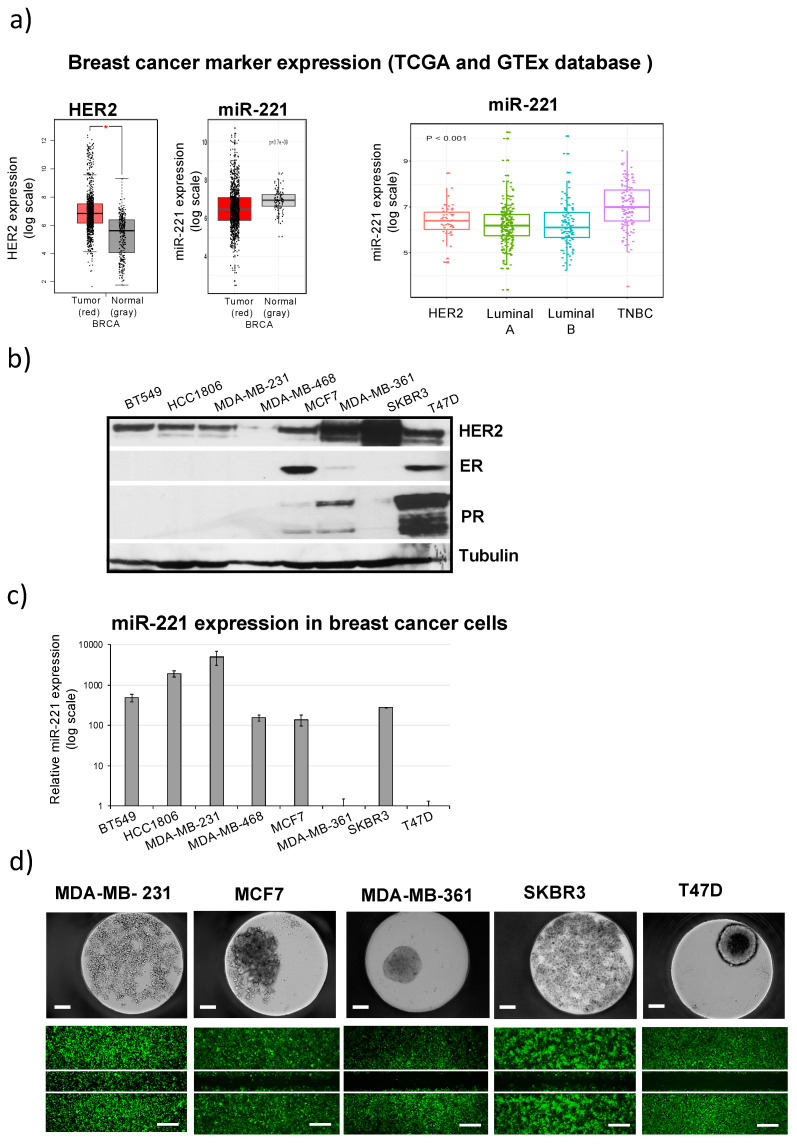
miR-221 expression in different breast cancer cell types including (**a**) TCGA database analysis of breast cancer samples for differential marker expression comprising HER2 (* *p*-value < 0.05) and miR-221 (*p*-value = 3.7 × 10^−9^) analysis and including tumor tissue (red) to normal tissue (gray) comparison (left part), GTEx database analysis for miR-221 expression including HER2+, Luminal A/B and TNBC subgroups (right part), (**b**) different breast cancer cell lines were analyzed for HER2, ER and PR expression and (**c**) miR-221 expression levels relative to the T47D settled as control (as 1) and normalized to RNU44 in qRT-PCR analysis, (**d**) example of five different breast cancer cell lines (MDA-MB-231, MCF-7, MDA-MB-361, SKBR3 and T47D) with constitutive lentiviral-GFP expression (24 h upon wound healing) indicated that higher migration capacity correlates with higher miR-221 expression level and shows inability for 3D-culture aggregation to generate spheroid formation, scale bar: 100 µm (3D) and 25 µm (migration).

The miR-221 and HER2 boxplots were generated using GEPIA interactive web server [18] from publicly available RNA sequencing expression databases including the TCGA and the GTEx projects (Figure 1a). The in silico analysis showed significant difference in miR-221 expression in comparison to normal adjacent tissue samples (Figure 1a, left) in agreement with previously published in house analysis [10]. Within TCGA database 512 samples were analyzed for miR-221 expression including different breast cancer subgroups: HER2+, Luminal A, Luminal B and TNBC (Figure 1a, right). Higher expression levels of miR-221 were shown for TNBC group and HER2+ in comparison to Luminal A and Luminal B groups (Figure 1a, right). Similarly, different miR-221 expression levels were detected in different breast cancer cell lines characterized according to their HER2, ER and PR protein status including TNBC cell lines (BT549, HCC1806, MDA-MB-231 and MDA-MB-468, Figure 1b,c). miR-221 expression levels were very high (1000-fold) for TNBC (BT549, HCC1806, MDA-MB-231) cell lines and high (100-fold) for MDA-MB-468 (TNBC), MCF7 (ER+), or SKBR3 (HER2 amplification+) cell lines when analyzed and compared to T47D cells, representing miR-221 low expressing cell line and used as control for miR-221 quantification in Figure 1c.

### 2.2. High miR-221 Expression Levels in Breast Cancer Cells Correlate with Low Attachment, Low Adhesion and High Migration Activity

Spheroid formation of five different breast cancer cell lines (Figure 1d) showed an association with miR-221 expression levels using an established 3D-microtissue platform [19]. The 100-fold higher miR-221 expression in MCF7 and SKBR3 cells (when compared to T47D), as well as 1000-fold higher miR-221 expression (Figure 1c) detected in MDA-MB-231 cells correlated with reduced capacity to form spheroids. Wound healing assays (Figure 1d) showed that high migration capacity associates with high miR-221 expression levels and the highest migratory activity was seen for MDA-MB-231 cells that express the highest levels of miR-221. In conclusion, the analysis of different breast cancer cell lines showed that high miR-221 expression levels (including TNBC and HER2 + cell lines) strongly correlate with breast cancer cell migration capacity and inhibit self-aggregation properties to form spheroids (Figure 1).

### 2.3. Breast Cancer 3D-Spheroid Analysis Using MEK1 Inhibitor (TAK-733) in Combination with Irradiation

**Figure 2:**
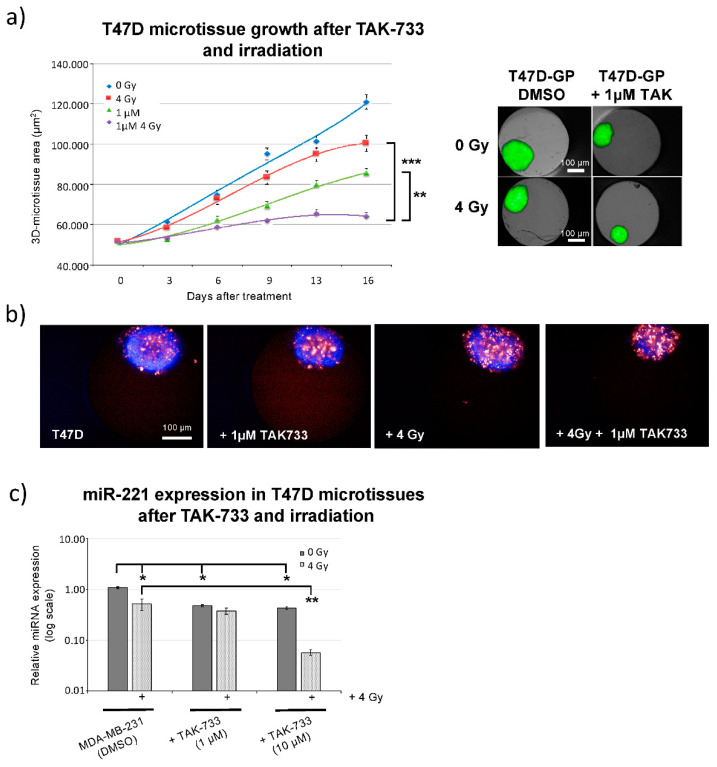
Growth analysis of T47D cells upon treatment with MEK1 inhibitor (TAK-733) and irradiation. (**a, left part**) GFP plot (area in µm^2^) quantification of spheroid growth delay assay at indicated time points, after 1 µM TAK-733 (MEK1i) treatment and 4 Gy irradiation at day 0 [normalization used between different wells and compared to control (1% DMSO) spheroids at day 0 and sham (0 Gy) irradiation, data are averages ± SD, *n* = 4; (*t*-test) ** *p*-value < 0.01, *** *p*-value < 0.001]. (**a**, **right part**) Example of Operetta bright-filed combined with GFP detection in breast cancer 3D-T47D-GP spheroids 9 days after indicated treatments including 4 Gy irradiation and 1 µM TAK-733 treatment (scale bar: 100 µm). (**b**) Example of T47D-GP spheroids stained with Hoechst 33342 (blue fluorescence—living cell fraction) and with DRAQ7 (red fluorescence—dead cell fraction), 9 days after 1 µM TAK-733 and 4 Gy irradiation (scale bar: 100 µm). (**c**) miR-221 expression (3 days) after 1 µM and 10 µM TAK-733 treatment and quantified by qRT-PCR upon 0 Gy and 4 Gy irradiation and normalized to sham (0 Gy) control 3D-T47D-GP (containing 1% DMSO) and RNU44 as endogenous miRNA control, [*n* = 2; ± SD; (*t*-test) * *p*-value < 0.05, ** *p*-value < 0.01].

It was previously shown that miR-221 expression is dependent on the oncogenic RAS-RAF-MEK pathway activity [13]. Therefore, the MEK1 inhibitor (TAK-733) was analyzed for synergistic effects with irradiation using our lately developed 3D microtissue assay platform [19] with T47D cells (Figure 2a). TAK-733 inhibited T47D-3D-spheroid growth up to 16 days of analysis (Figure 2a) and the combination of compound and 4 Gy treatment showed an additional significant growth delay (*p* = 0.0023, Figure 2a).

These data were supported using living (Hoechst 33342) and dead (DRAQ7) cell stainings (Figure 1b), showing increased fraction of dead (red) cells upon combined TAK-733 and 4 Gy irradiation treatment. TAK-733 (MEKi) or 4 Gy irradiation alone resulted in detection of more dead cells compared to sham irradiated (DMSO treated) controls and lower numbers of living (“blue”) cells were identified. Spheroids with disseminated borders were detected 9 days upon indicated treatments (Figure 1b). Additionally, the influence of TAK-733 MEK1 inhibitor on miR-221 expression was analyzed from spheroids (three days upon treatment) with 1 µM and 10 µM TAK-733 concentration combined with 4 Gy irradiation. TAK-733 (10 µM) significantly inhibited miR-221 expression when used in combination with 4 Gy irradiation (Figure 1c). These data revealed that MEK1 inhibitor treatment combined with irradiation could significantly decrease the growth of 3D breast cancer microtissues including reduction in miR-221 expression.

### 2.4. MEK1 Inhibitor (TAK-733) Impedes Breast Cancer Cell Migration in Combination with Irradiation

**Figure 3:**
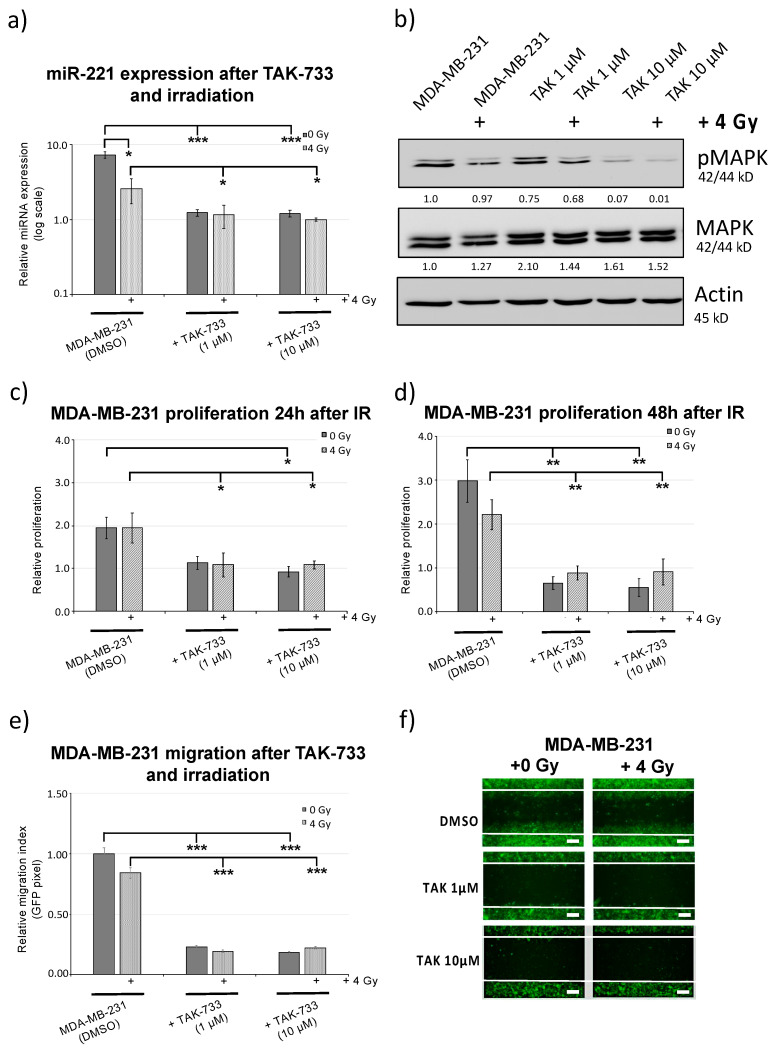
miR-221 expression and migration analysis upon MEK1 inhibitor treatment combined with 4 Gy irradiation. (**a**) miR-221 expression (48 h) after 1 µM and 10 µM TAK-733 treatment and quantified by qRT-PCR upon 0 Gy (dark gray) and 4 Gy (light gray) irradiation and normalized to sham (0 Gy) control MDA-MB-231 cells (containing 1% DMSO) and RNU44 as endogenous miRNA control, [*n* = 3; ± SD; (*t*-test) * *p*-value < 0.05; *** *p*-value < 0.001]. (**b**) Protein analysis of MAPK phosphorylation and total MAPK in MDA-MB-231 cells (24 h) upon 1 µM and 10 µM treatment with TAK-733 (MEK1i) and 4 Gy irradiation. (**c**) Relative proliferation of MDA-MB-231 cells 24 h and (**d**) 48 h upon 1 µM and 10 µM treatment with TAK-733 (MEK1i) and 4 Gy irradiation and normalized to sham (0 Gy) control MDA-MB-231 cells at 0 time point (immediately after irradiation and 1% DMSO treatment), [*n* = 3; ± SD; (*t*-test) * *p*-value < 0.05; ** *p*-value < 0.01]. (**e**) Quantification of the migration capacity of MDA-MB-231 control cells (dark gray) and upon combined TAK-733 and 4 Gy irradiation treatment (light gray) using the Image Colour Analyser (determines the number of green pixels within the gap) after 48 h and TAK-733 compound concentration and irradiation indicated, [*n* = 3; ± SD; (2 way ANOVA) *p*-value: *** < 0.001]. (**f**) Exemplary wound healing of MDA-MB-231-GP cells (48 h) after TAK-733 treatment (1 µM and 10 µM) including 0 Gy and 4 Gy irradiation (scale bar: 25 µm).

3D-microtissue assays were not possible for MDA-MB-231 cells naturally and highly expressing miR-221 and showing low self-aggregation activity (Figure 1d). The influence of TAK-733 MEK1 inhibitor on miR-221 expression was further analyzed using MDA-MB-231 breast cancer cells, 48 h after treatment with 1 µM and 10 µM TAK-733 combined with 4 Gy irradiation. TAK-733 (1 µM) significantly inhibited miR-221 expression when used in combination with 4 Gy irradiation (Figure 3a). The specificity of MEK1 inhibitor was confirmed by analyzing ERK1/2 phosphorylation status within MDA-MB-231 cells and by applying 1 µM and 10 µM TAK-733 (Figure 3b and Supplemental Figure S1) for 24 h and 48 h. Remarkably, the effect on ERK1/2 (42/44 MAPK) phosphorylation was slightly decreased (not significantly) with 1 µM TAK-733, but significantly decreased phosphorylations were detected with 10µM TAK-733 (Figure 3b). Proliferation data analysis showed strong cytostatic activity for TAK-733 including 24 h and 48 h time point of analysis (Figure 3c,d). Additionally, these data indicate potential usage of miR-221 expression changes as therapeutic biomarker for MEK1-targeted treatment activity. Along with miR-221 reduction, TAK-733 treatment significantly reduced the migration activity of MDA-MB-231 cells and the migration capacity remained significantly reduced when combined with 4 Gy irradiation 24 h and 48 h upon treatment. Significant changes to sham irradiated controls (Figure 3e,f) were evident for 1 µM TAK-733, previously confirmed to influence miR-221 expression (Figure 3a) and proliferation.

### 2.5. MEK1 Inhibitor (TAK-733) Combined with Irradiation Inhibits ZEB1 (EMT Marker) Activity

**Figure 4:**
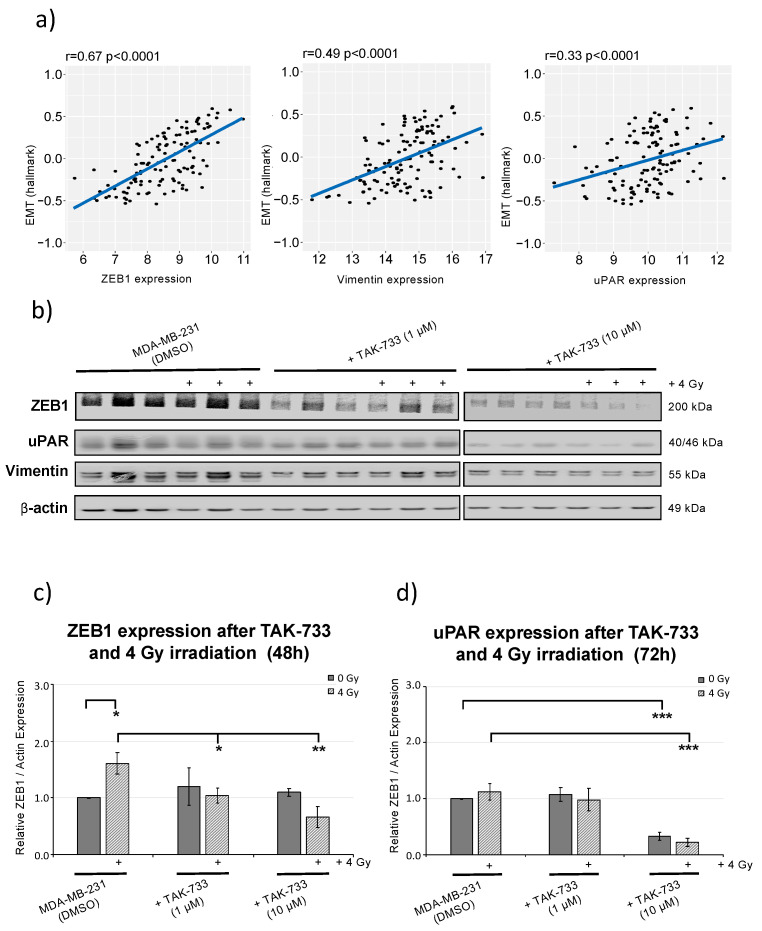
ZEB1, Vimentin and uPAR expression analysis upon MEK1 inhibitor treatment combined with 4 Gy irradiation. (**a**) scatterplot of EMT hallmark module vs. gene significance for ZEB1, Vimentin and uPAR expression from 123 TNBC patients within TCGA cohort. (**b**) Protein analysis of ZEB1, Vimentin and uPAR in MDA-MB-231 cells (48 h) upon 1 µM and 10 µM treatment with TAK-733 (MEK1i) and 4 Gy irradiation (three independent cell lysates were loaded in parallel, *n* = 3). (**c**) Relative quantification of combined TAK-733 and 4 Gy irradiation on ZEB1 and (**d**) uPAR expression to b-actin and normalized to MDA-MB-231 sham irradiated control cells (treated with DMSO), [*n* = 3; ±SD; (2 way ANOVA) * *p*-value < 0.05; ** *p*-value < 0.01; *** *p*-value < 0.001].

In order to find the genes which were highly associated with EMT hallmarks and potentially influence the mode of cell migration, patient data sets were analyzed for ZEB1, Vimentin and uPAR expression (Figure 4a).

The ZEB1 expression showed the highest gene significance with EMT module analysis (r = 0.067; *p* < 0.0001) from 123 TNBC patients within TCGA cohort. ZEB1 transcription factor expression (a central EMT regulator) was shown to be significantly induced after irradiation alone (Figure 4b,c and Figure S2) which supports radiation resistance for MDA-MB-231 cells, providing stable migration activity, seen in the controls (DMSO) upon 0 Gy and 4 Gy irradiation (Figure 3c,d). Finally, the results revealed that MEK1 inhibitor (when combined with radiation) significantly reduces the migratory potential of MDA-MB-231 breast cancer cells including reduction of miR-221 expression (Figure 3a) and corresponding downstream ZEB1 and uPAR (EMT) marker expression (Figure 4b–d).

### 2.6. Modulation of miR-221 Expression Alters Migration Capacity

**Figure 5:**
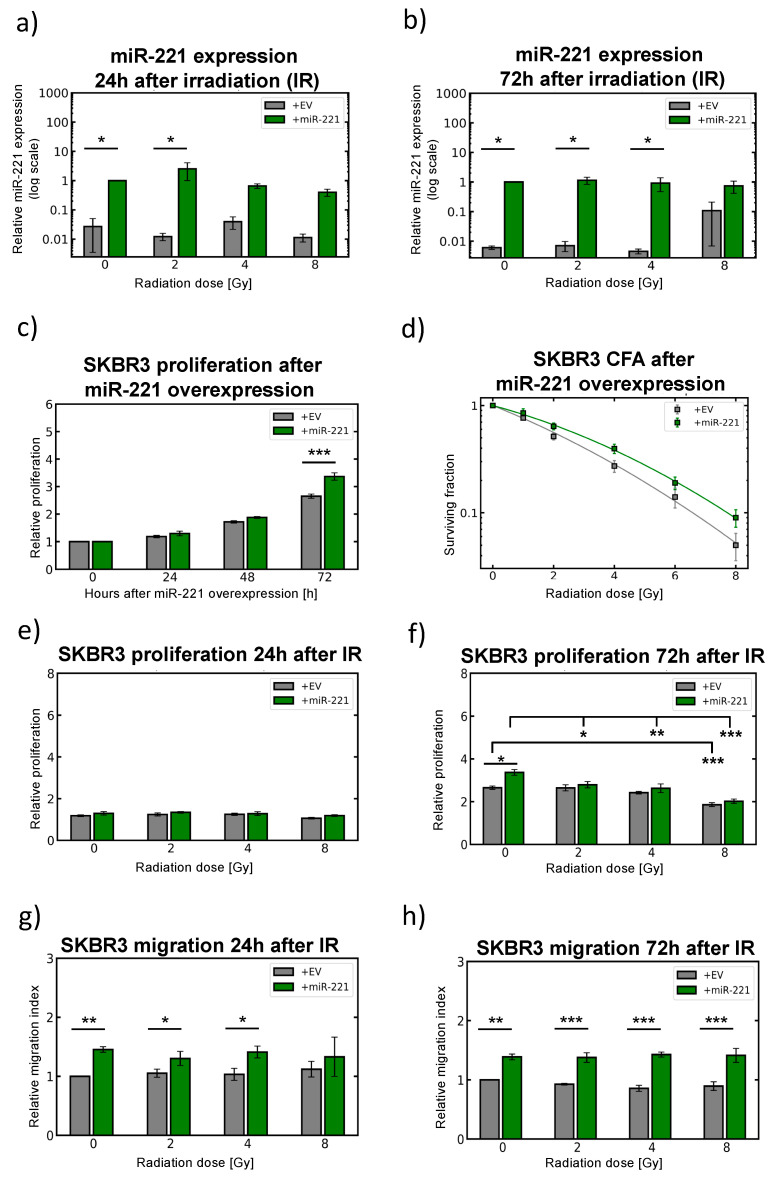
miR-221 overexpression influences SKBR3 migration activity and response to irradiation. (**a**) miR-221 lentiviral overexpression (green) quantified by qRT-PCR (24 h) and (**b**) 72 h upon 0, 2, 4 and 8 Gy irradiation compared to (EV) control SKBR3 (grey) cells and normalized to sham (0 Gy) SKBR3 (EV) cells and RNU44 as endogenous miRNA control, [*n* = 3; ±SEM; (ANOVA and post-hoc test) * *p*-value < 0.05]. (**c**) SKBR3 cell proliferation upon miR-221 overexpression (green) with significant changes to corresponding SKBR3 (EV) control (gray) cells at 0 time point (before irradiation) and additional time points 24 to 72 h without irradiation, [*n* = 3; ±SEM; (ANOVA and post-hoc test) ** *p*-value < 0.01]. (**d**) Clonogenic survival is increased upon miR-221 overexpression in SKBR3 cells when the cells were irradiated with 2, 4, 6 and 8 Gy. After 10 days colonies bigger than 50 cells were counted, [*n* = 4; ±SEM] (**e**) Relative proliferation index of SKBR3 miR-221 overexpressing cells (green) 24 h and (**f**) 72 h after 0 Gy to 8 Gy irradiation and normalized to sham (0 Gy) empty virus (EV) control SKBR3 cells (gray), [*n* = 3; ±SEM; (ANOVA and post-hoc test) * *p*-value < 0.05; ** *p*-value < 0.01; *** *p*-value < 0.001]. (**g**) Migration activity (wound healing capacity) of control (EV) cells (gray) and miR-221 overexpressing SKBR3 cells (green) 24 h and (**h**) 72 h after irradiation treatment. Quantification proceeded by the Image Colour Analyser (determines the number of green pixels within the gap) after irradiation doses indicated, [*n* = 3; ±SEM; (ANOVA and post-hoc test) * *p*-value < 0.05; ** *p*-value < 0.01; *** *p*-value < 0.001].

**Figure 6:**
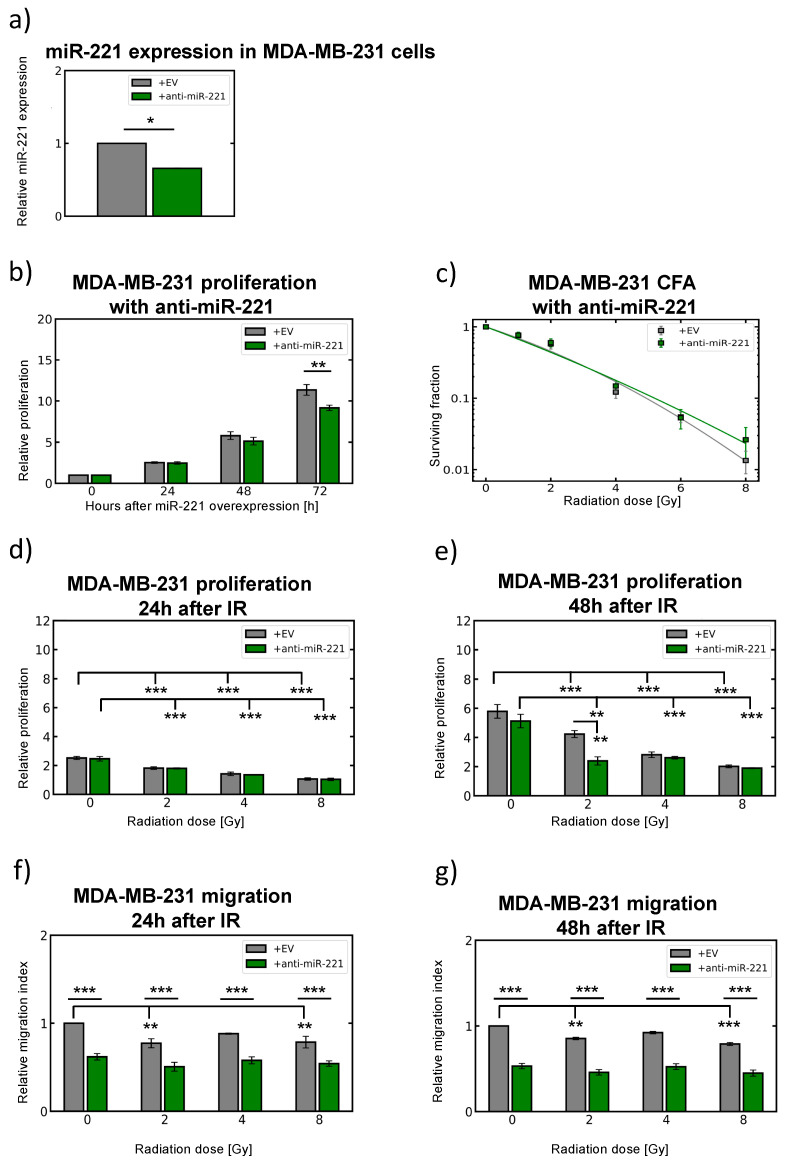
miR-221 knock-down (anti-miR-221) influences MDA-MB-231 migratory response to irradiation. (**a**) anti-miR-221 (green) quantified by qRT-PCR (72 h) upon transduction and compared to empty virus (EV) control (gray) MDA-MB-231 cells and RNU44 as endogenous miRNA control, [*n* = 3; ±SEM; (*t*-test) *p*-value: * <0.05]. (**b**) MDA-MB-231 cell proliferation upon miR-221 knock-down (anti-miR-221) with significant changes to corresponding (EV) control treatment at 0 time point (before irradiation) and additional time points 24 to 72 h without irradiation, [*n* = 3; ±SEM; (ANOVA and post-hoc test) ** *p*-value < 0.01]. (**c**) Clonogenic survival upon miR-221 knock-down (anti-miR-221) in MDA-MB-231 cells when the cells were irradiated with 2, 4, 6 and 8 Gy. After 10 days colonies bigger than 50 cells were counted, [*n* = 3; ±SEM], (**d**) Relative proliferation index of MDA-MB-231 anti-miR-221 cells (green) 24 h and (**e**) 48 h after 0, 2, 4, and 8 Gy irradiation and normalized to sham (0 Gy) control (EV) MDA-MB-231 cells (gray) at 0 time point (before irradiation) [*n* = 3; ±SEM; (ANOVA and post-hoc test) ** *p*-value < 0.01; *** *p*-value < 0.001]. (**f**) Migration activity (wound healing capacity) of control (EV) (gray) and anti-miR-221 MDA-MB-231 cells (green) 24 h and (**g**) 48 h after irradiation treatment. Quantification proceeded by counting the number of green pixels within the gap excluding background fluorescent fluctuations after irradiation doses indicated, [*n* = 3; ±SEM; (ANOVA and post-hoc test) ** *p*-value < 0.01; *** *p*-value < 0.001].

According to different levels of miR-221 expression in breast cancer cell lines (Figure 1c), SKBR3 cells were used to overexpress miR-221 and MDA-MB-231 cells (1000-fold naturally overexpressing miR-221) were used for knock-down experiments by lentiviral approach (Figure 5 and Figure 6). Initially, the migration capacity of SKBR3 cells was analyzed 24 h following irradiation (Figure S3). Compared to sham irradiated controls (0 Gy, Figure S3a) higher radiation doses (4 Gy and 8 Gy) slightly induced the migratory phenotype of SKBR3 cells 24 h upon treatment. This was in line with apoptosis induction and increased caspase 3/7 enzyme activity detection after irradiation treatment, whereas cell viability was not dramatically decreased 24 h upon irradiation (Figure S3). To confirm that migration activity is linked with higher miR-221 expression, miR-221-overexpressing SKBR3 cells were analyzed in parallel with control cells (containing empty virus (EV) control, Figure 5). miR-221-overexpressing SKBR3 cells showed (10 fold and 100 fold) higher miR-221 expression levels when compared to non-irradiated (0 Gy) empty vector (EV) control cells, 24 h (** *p* < 0.0100) to 72 h after treatment with 2 Gy, 4 Gy and 8 Gy (*** *p* < 0.0001, ANOVA between SKBR3 (EV) and +miR-221 overall, Figure 5a,b). Additionally, miR-221 expression levels were not changing dramatically 24 h and 72 h upon 4 Gy (and 8 Gy) irradiation (Figure 5a,b), showing that irradiation alone is not significantly changing expression of miR-221. Slight miR-221 reduction was detected upon 8 Gy irradiation (Figure 5a), but the levels of miR-221 were still significantly higher in miR-221-overexpressing SKBR3 cells when compared to corresponding control (EV) cells 24 h and 72 h after irradiation treatment (Figure 5a,b). Higher miR-221 expression induces higher proliferation capacity of SKBR3 miR-221-overexpressing cells (Figure 5c) 72 h after seeding for the relative proliferation index analysis.

Colony formation assays confirmed an increased radiation resistance of SKBR3 cells overexpressing miR-221 ten days upon irradiation (*** *p* < 0.0030, ANOVA between SKBR3 (EV) and +miR-221 overall, Figure 5d). Additionally, 24 h post-irradiation, no effects on proliferation were detected either in EV control cells nor in miR-221-overexpressing SKBR3 cells (Figure 5e), whereas significant reduction in SKBR3 cell proliferation was detected 72 h upon 8 Gy irradiation independent of miR-221 overexpression (Figure 5f). Correspondingly, significant upregulation in migration capacity was detected for miR-221-overexpressing SKBR3 cells, 24 h after irradiation treatment (** *p* < 0.0106, Figure 5g), that retained to be notably higher even 72 h upon 2 Gy, 4 Gy and 8 Gy irradiation (*** *p* < 0.0003, ANOVA between SKBR3 (EV) and +miR-221 overall, Figure 5h). These data confirm high migration activity of miR-221-overexpression in SKBR3 cells upon irradiation treatment.

Additionally, MDA-MB-231 cells (naturally expressing 1000-fold high levels of miR-221) were used for anti-miR-221 (knock-down) experiments including radiation treatment (Figure 6). Reduction in miR-221 expression (Figure 6a), when compared to empty vector (EV), confirmed reduced proliferation capacity for anti-miR-221 MDA-MB-231 cells (Figure 6b), 72 h after seeding for relative proliferation index analysis. As the knockdown effect was lowering miR-221 levels (Figure 6a), significant radiosensitization effects of anti-miR-221 treatment were not detected in colony formation assays (Figure 6c), as probably much higher reduction of miR-221 expression level is required. Significant reduction in MDA-MB-231 cell proliferation was detected 24 h to 48 h upon 8 Gy irradiation independent of miR-221 knock-down (Figure 6d,e), including considerably reduced migration of MDA-MB-231 cells by anti-miR-221, when compared to corresponding (EV) controls upon different radiation doses (2 Gy, 4 Gy and 8 Gy, *** *p* < 0.0001, ANOVA 24 and 48 h between MDA-MB-231 (EV) and +miR-221 overall, Figure 6f,g). The data observed in both breast cancer cell lines suggested that higher miR-221 expression levels significantly influence cellular migration capacity and EMT turnover. Moreover, miR-221 expression levels were very stable upon radiation treatment.

## 3. Discussion

Taken together, the results presented here showed that novel therapy design including MEK1 inhibitor (TAK-733) can be seriously considered in the future for the heavily pre-treated advanced breast cancers (e.g., TNBC and HER2+), while efficiencies may depend on different drug combinations and respective time and dose points. TAK-733 (as 1 µM) showed significant effects on miR-221/ZEB1 reduction and breast cancer cell proliferation and migration capacity (Figure 3 and Figure 4). In the future the clear and precise low concentration selection of substances without toxic side effects in combination with radiation therapy would be of high importance due to the benefits for the patients [4,20].

**Figure 7:**
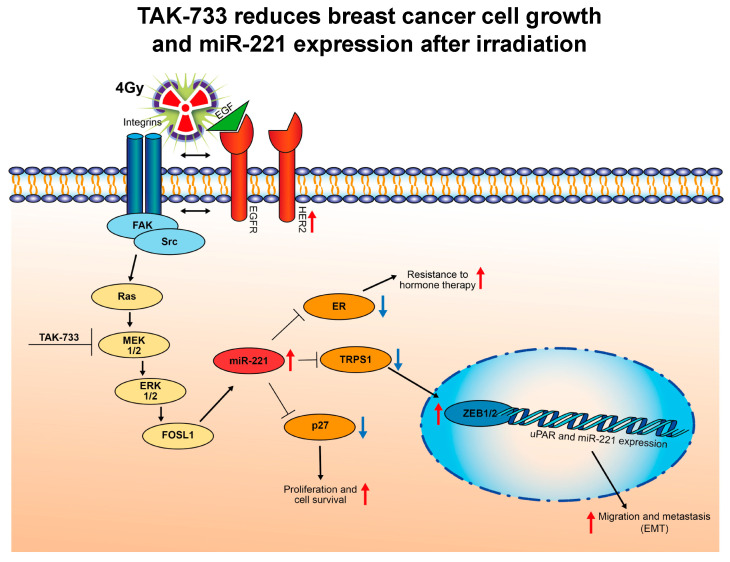
Proposed novel mechanism of TAK-733 (MEKi) action influencing activity and expression of miR-221 when combined with irradiation.

Compounds such as the inhibitors of mitogen-activated protein kinase kinase (MAPKK or MEK1) demonstrate promising effects on cancer cells [21,22]. MEK1 as part of the RAF/MEK/ERK signaling pathway is documented as important transmitter of extracellular signals from receptors on plasma membrane to the cell nucleus (Figure 7). An effective inhibitor for this signaling pathway is the selective, ATP-non-competitive MEK1 inhibitor represented here as TAK-733. The small molecule inhibitor binds to the allosteric site of MEK1 and shows antitumor activity in many mouse xenograft models, including a model for breast cancer [23]. Despite the fact, that the TAK-733 homologue Trametinib is currently FDA-approved for treatment of melanomas and is further in frequent use regarding phase 1 and 2 clinical trials (www.cancer.gov), the mechanisms behind the synergistic effects with radiation treatment still remain to be fully elucidated. 

The RAS-RAF-MEK-MAPK pathway is altered in ~40% of all human cancers, mainly due to mutations in BRAF (~10%) and its upstream activator RAS (~30%) [24,25]. Despite initial high response rates with targeted therapies against RAF and MEK, these therapies are limited due to the emergence of drug resistance and, over the last years, a number of acquired resistance mechanisms were discovered (reviewed in [26,27]). In recent years, a number of studies suggested that drugs targeting epigenetic alterations could be applied in synergy with other anticancer therapies or, importantly, in reversing acquired therapy resistance (reviewed in [25,28,29,30]). Therefore, combination therapies not only result in higher overall response rates and prolonged time to disease progression when compared with single agents, but could also offer an option for women with anthracycline- and taxane-pre-treated metastatic breast cancer [4,31]. Cardiotoxicity is a well-known side effect of anthracyclines (e.g., Doxorubicine) and the mechanisms leading to this phenomenon include high expression of miR-221 [32,33].

It is known that through an ERK1/2 phosphorylation MEK1 is able to activate many transcription factors and processes such as cell growth, migration and epithelial-to-mesenchymal transition (EMT) [34] as indicated in Figure 7. The miR-221 expression is dependent on the oncogenic RAS-RAF-MEK pathway activation (Figure 7) and potentially influences different cellular processes including EMT [12,13]. In parallel by regulating the level of miR-221 expression after combined MEK1 and 4 Gy irradiation, additional downstream targets may be affected (Figure 7), as e.g., TRPS1 previously shown to regulate ZEB1/2 transcription factor activity [12]. Data presented here highly suggest that MEK1 inhibitor (TAK-733) in combination with radiation may be an effective and advantageous treatment, especially for differentially miR-221-overexpressing cells (e.g., TNBC-very high and HER2 + high miR-221 expressing cells) that are recognized as highly migrative and resistant to radiation treatment alone.

Overexpression of miR-221 can be effectively inhibited using antisense oligonucleotides. However, several challenges regarding the stability and delivery strategies of the anti-miRs still need to be answered before they can be useful as therapeutics. In parallel, novel drugs known to prevent cardiovascular disease by lowering the levels of miR-221 are highly appreciated for future cancer treatments. With our work presented here, we have demonstrated that MEK1 inhibitor (TAK-733) significantly reduces miR-221 expression. By lowering miR-221 expression, migrative and proliferative features of breast cancer cells were inhibited and prevention of cardiotoxicity as a major side effect of breast cancer radiation therapy [35] is conceivable.

## 4. Materials and Methods

### 4.1. Omics Data Analysis Using Web Databases with Clinical Follow-Up 

HER2 and miR-221 expression markers were generated by GEPIA web server during 2019 and 2020 [18] analyzing the RNA sequencing expression data from 1085 breast tumors (BRCA) and 291 normal samples from the TCGA and the GTEx projects (Figure 1a). Additionally, RNA-Seq, stem-loop miRNA-seq and clinic datasets from patients with BRCA (breast cancer) were downloaded from the UCSC Xena browser (https://xenabrowser.net/). Box plots of miR-221 expression levels (Figure 1a) were created using ggplot2 (https://xenabrowser.net/) by analyzing 515 samples in total and including HER2+ (52 samples), Luminal A (223 samples), Luminal B (121 samples) and TNBC (119 samples). The data for EMT and the target gene (ZEB1, Vimentin and uPAR) analysis (Figure 4a) were retrieved from 123 TNBC patients within TCGA cohort in 2019 and 2020. The correlation was calculated with Pearson’s coefficient. The EMT score was obtained with the single-sample GSEA method analysis performed with “GSVA” package in R software (version: 3.63) [36].

### 4.2. Growth and Maintenance of Cell Lines and 3D-Microtissue Generation

The breast cancer cell lines BT549, HCC1806, MDA-MB-231, MDA-MB-468, MCF7, MDA-MB-361, SKBR3, and T47D (acquired from ATCC, Gaithersburg, MA, USA or DSMZ GmbH, Braunschweig, Germany collections) were maintained in DMEM (Dulbecco’s Modified Eagle Medium) supplemented with 10% FCS and non-essential amino acids (Sigma Aldrich, St. Lois, MO, USA) when necessary or in RPMI 1640 (Roswell Park Memorial Institute) medium supplemented with 10% FCS and human insulin (10 µg/mL). The human embryonic kidney HEK293T cells (from DSMZ GmbH collection) were used for lentivirus productions and grown in DMEM medium with 10% FCS. Cultivation was performed under standard conditions in water humified 37 °C incubator with 5% CO_2_, either for 2D or 3D cell analysis. Cell lines were checked for mycoplasma contamination using the MycoAlert Detection Kit (Lonza Group Ltd., Basel, Switzerland) and their identity verified by genetic profiling using the PowerPlex^®^ 16 System (Eurofins/MWG Operon, Munich, Germany).

### 4.3. Lentivirus Production and Infection of Breast Cancer Cell Lines

Replication-defective lentiviral particles were produced by transient co-transfection of HEK293T cells in a 10 cm petri dish using Lipofectamine 2000 (Life Technologies, Carlsbad, CA, USA) according to the manufacturer’s instructions. Transfection mix contained 16 µg, 8 µg and 4 µg of packaging plasmids pMDLg/pRRE, pRSV.Rev and pMD2.G (a kind gift from D. Trono, École polytechnique fédérale de Lausanne, Switzerland) and 8 μg of lentiviral transduction vector pGreenPuro (pGP) expressing copGFP (System Biosciences, Palo Alto, CA, USA) used as empty vector (EV) control for lentiviral infections or corresponding miR-221 overexpression (pMIRNA-221, Cat. No. MIRH221-PA-1-GVO-SBI; Biocat, Heidelberg, Germany) or anti-miR-221 knock-down vector (pmiRZIP-221, Cat. No. MZIP221-PA-1-GVO-SBI; Biocat; Heidelberg, Germany). The virus particles were harvested 48 h after transfection of 293T cells cultivated in water humified 37 °C incubator with 5% CO_2_, cleared and concentrated as previously described [37,38,39]. According to virus titer determination virus productions ranged between 10^8^ to 10^9^ TU/mL and viral infection of breast cancer cells was performed using previously described protocols [37,38,39,40,41]. Briefly, 2 × 10^5^ cells per well were infected with 4 × 10^5^ TU/mL (2 MOI) for 16–24 h in water humified 37 °C incubator with 5% CO_2_. After infection the cells were washed of virus (2xPBS) and cultivated further in corresponding medium. Three days after infection, GFP expression was monitored and cells were seeded in petri dishes for subsequent analysis. Additionally, GFP expressing cells were seeded in 96-well GravityTRAP ULA plates (InSphero AG, Schlieren, Switzerland and Perkin Elmer, Waltham, MA, USA) for 3D microtissue assay analysis or in 12-well Ibidi chambers (Ibidi, Munich, Germany) for wound healing (migration) assay analysis and treated as described below.

### 4.4. Generation of Tumour 3D-Microtissues and Treatment with Compounds and Irradiation

3D microtissues were generated using 500 cells (per well) showing adequate growth kinetics and low interwell variations (bellow 10%). Cell density in media was estimated using a hemocytometer (Z1 Coulter, Beckman, Brea, CA, USA). 3D-microtissues were formed by seeding T47D, MDA-MB-361, MCF7, SKBR3 and MDA-MB-231 cells into the GravityTRAP plate and maturing them for 3 days followed by treatment with 1µM or 10µM compound (TAK-733) where necessary. Compound concentrations were made that 1% of the total volume per well consists of compound-DMSO dilutions. After one day of substance treatment (defined as day 1 of treatment), tissues were sham irradiated (0 Gy) or irradiated with a single acute dose of 4 Gy with a X-Strahl RS225 (X-Strahl LTD, Camberlay, UK) with delivered dose rate of 0.82 Gy/min and including a 3mm aluminum filter. The exposed and sham irradiated 3D-microtissues were subsequently incubated at 37 °C with 5% CO_2_ for indicated time points. The experiment was repeated for each dose in quadruplicates and in three independent experiments (*n* = 3).

Growth of 3D-microtissues was followed in assay plates for 16 days or labelled with 1µM DRAQ7 (red) or 0.1 µM Hoechst 3322 (blue) for 20min at room temperature and then further processed using Operetta^®^ High Content Imaging System (Perkin Elmer, Waltham, MA, USA) device. Images from a single plate were acquired (when necessary) with the GFP (green), Hoechst 3322 (blue), DRAQ7 (red) and Brightfield channels using the 10 × NA objective in wide field mode. Direct quantification of 3D-microtissue fluorescent area using a high imaging platform accelerates captures the full range of microtissue phenotypes during analysis using Harmony^®^3.1 High Content Imaging and Analysis Software (Perkin Elmer, Waltham, MA, USA). The Find Image Region Building Block was applied to the GF (green), red or Hoechst channel to detect the microtissues in the well. As a next step, the Calculate Morphology Building Block was added to calculate the tissue area (µm^2^) as the final readout for green channel. For statistical analysis the Student’s *t*-test was used.

### 4.5. RNA Isolation for miRNA Expression Analysis

Total RNA was isolated from each of the breast cancer cell lines or after viral transductions (including SKBR3 and MDA-MB-231 cells) and upon TAK-733 and irradiation treatment when indicated. The cells were pelleted by centrifugation at 1500 rpm for 5 min and washed with 1 mL Dulbecco’s phosphate-buffered saline (PBS) without MgCl_2_ and CaCl_2_ (Invitrogen, Thermo Fisher, Waltham, MA, USA). Small RNAs (<200 nucleotides) were isolated from the cells using the miRNA Tissue isolation kit and Maxwell16 device (Promega, Madison, WI, USA) following the protocol for total RNA isolation. The quantity and quality of the total RNA including miRNA fraction was measured with the Nanodrop spectrophotometer (PeqLab Biotechnology, Erlangen, Germany).

### 4.6. TaqMan-miRNA Assays and Data Analyses

Specific single TaqMan—miRNA assays (Applied Biosystems, Thermo Fisher, Waltham, MA, USA) were used for miR-221 expression analysis (Cat.Nr. 4427975; Assay ID 000524) in total RNA isolations from cell culture pellets according to previously described protocols. Quantitative RT-PCR was performed using the StepOnePlus Detection System (Thermo Fischer, Waltham, MA, USA). The relative expression values of specific miR-221 were calculated by using the 2^–ΔΔCT^ method [38] and normalized to the control miRNA (RNU44, # 4427975; Assay ID 001095) [10] and relative to the T47D or SKBR3 (EV) or MDA-MB-231 (EV) as control cells (used as calibrator) alone or after subsequent lentiviral transductions or compound and radiation treatment. All reactions were performed in duplicates and three independent experiments (*n* = 3) were conducted.

### 4.7. Western Blot Analysis

The isolation of proteins, immunoblotting and quantifications were performed as previously described [10,42]. The respective target proteins regulated by the miRNAs were detected with the following primary antibodies: HER2 (A0485, DAKO, Glostrup, Denmark), MAPK (9101) and phospho-MAPK (9102, Cell Signaling Techn., Danvers, MA, USA), ZEB1 (HPA027524; Atlas Antibodies, Bromma, Stockholm, Sweden) a kind gift from Prof. K.P. Janssen (Department of Surgery, Technical University of Munich, Munich, Germany), Vimentin (ab92547; Abcam, Cambridge, UK), uPAR (clone IID7) a kind gift from Prof. M. Schmitt and Prof. V. Magdolen (Clinical Research Unit, Department of Obstetrics and Gynecology, Technical University of Munich, Munich, Germany) [43] and tubulin or actin as a loading control (A5441, Sigma, St. Louis, MO, USA). Estrogen receptor (ER), progesterone receptor (PR) were used as previously published [44]. The following peroxidase-conjugated secondary antibodies were used: anti-rabbit (A16096) and anti-mouse (16066) (Invitrogen, Carlsbad, CA, USA).

### 4.8. Cell Viability and Caspase 3/7 Activity Assay

Cell viability and caspase 3/7 activity was determined using corresponding luminescence assays (CellTiterGlo Cat.Nr. G7570 and Caspase-Glo 3/7 assay Cat. Nr. G8090) according to the manufacturer’s protocols (Promega, Madison, WI, USA). The SKBR3 and MDA-MB-231 cells were seeded at a concentration of 1 × 10^5^ cells per well in a 6-well tissue culture plates (when necessary) day before treatment with TAK-733, irradiated (0, 2, 4, 8 Gy) and incubated for 24, 48 or 72 h at 37 °C. Additionally, 1 × 10^5^ of SKBR3 (EV) controls in parallel with +miR-221 overexpression or 1 × 10^5^ MDA-MB-231 (EV) controls in parallel with +anti-miR-221 cells were seeded using 6-well tissue culture plates. After 24 h, cells were irradiated (0, 2, 4, 8 Gy) and immediately after irradiation (0 h) cells were counted using a hemocytometer (Z1 Coulter, Beckman, Brea, CA, USA) and counts were repeated 24, 48 and 72 h after irradiation to monitor cell proliferation after specific treatment. The measurements were performed in duplicates for three independent experiments (*n* = 3).

### 4.9. Migration Assay

Gap-closure (wound healing) was performed with stable lentiviral GFP-labelled cells according to previous publication [45]. For migration analysis of SKBR3-GFP cells, silicon grids (Ibidi, Munich, Germany) with 2 rectangular wells (500 µM gap) were placed (air bubble-free) in 12 well plates. For migration experiments including MDA-MB-231-GFP cells, silicon grids (Ibidi, Munich, Germany) with 12 rectangular wells (2 mm gap) were placed (air bubble-free) in 10 cm cell culture dishes. 60,000 of SKBR3 (EV) controls or +miR-221 and 40,000 of MDA-MB-231 (EV) controls or + anti-miR-221 were then seeded per well. For migration cell analysis upon combined compound and radiation treatment 100,000 of MDA-MB-231 (EV or +anti-miR-221) cells were seeded per well within 12 rectangular wells (2 mm gap). After cell attachment the medium was discarded and cells were pretreated with 1 or 10 μM of the MEK1-inhibitor TAK-733 (SeleckChem, Munich, Germany, Cat. No. S2617) or DMSO (Sigma-Aldrich, St. Louis, MO, USA) when necessary. In all experiments 24 h after cell plating the irradiation (0, 2, 4, 8 Gy) was performed as stated on the Figures and the silicon grids were carefully removed to generate a defined gap in the monolayer. Medium containing 1 or 10 μM TAK-733 or DMSO was added to the cells when necessary. Starting pictures (0 h) were taken immediately after grid removal and repeated after 24, 48 and 72 h to monitor migration.

For quantification Adobe Photoshop CS5 (Adobe Systems) and Image Colour Analyser program (developed by Marcus Vetter; source code available upon request) were used to quantify the migratory potential. Areas with green cells of the 0 h picture were subtracted from pictures of later time points and the green value of 2 mm gap area was measured (including the subtraction of fluorescent background signals). For quantification of MDA-MB-231 (EV) and +anti-miR-221, the number of green pixels were counted within the gap after processing using scharr algorithm to exclude fluorescent background fluctuations (by Christoph Herb, program code available on demand). All experiments were performed three times (*n* = 3) with five to seven technical replicates.

### 4.10. Statistics

For statistical analyses of the in vitro proliferation, caspase assays, 3D-microtissue and migration assays, as well as for Western blots, Student’s *t*-test was used. Data show the mean of independent biological experiments with the standard deviation (±SD). The two-sided paired, unpaired or the one-sample *t*-test were used for statistical analysis and a *p*-value ≤ 0.05 was deemed statistically significant, while a *p*-value < 0.01 was considered highly significant. Additionally, migration assays and western blots were confirmed with two-way ANOVA analysis. Since the ANOVA only provides information about significant differences in general, for example a general effect of irradiation or compound treatment, post hoc testing was performed to compare individual treatments. Bonferroni, Sidak or Dunnett multiple comparisons were done with GraphPad Prism5 to identify the individual significant differences. To analyze significant effects on specific marker proteins by the inhibitors alone irradiated compound-treated cells were compared to their irradiated control (4 Gy, DMSO) and the sham-irradiated compound-treated samples were compared to their sham-irradiated controls (DMSO). For the analysis of interaction effects between irradiation and inhibitor treatment 0 Gy treated samples were compared to the 4 Gy treated samples. A *p*-value < 0.05 was deemed statistically significant and *p*-values < 0.01 were considered to be highly significant.

## 5. Conclusions

The results presented here confirm that MEK1 inhibitor treatment combined with irradiation could significantly decrease the migratory potential of breast cancer cells including reduction in miR-221 and EMT (ZEB1) marker expression changes. By using MEK1 inhibitor in combination with radiation therapy for advanced breast cancer, the miR-221 holds great potential as therapeutic marker of metastatic breast cancer and as promising target for new drug developments. However, more insight into efficiency of miR-221 inhibition needs to be followed in the future.

## Supplementary Materials

The following are available online at https://www.mdpi.com/2072-6694/12/12/3760/s1, Figure S1. Protein analysis of MAPK phosphorylation and total MAPK in MDA-MB-231 cells. Western Blots for the proteins shown in Figure 3. MDA-MB-231 cells were treated with DMSO or compound TAK-733 at the concentrations indicated or additional 4 Gy irradiation and blots were probed with antibody for the protein indicated. Molecular weight markers are shown in the left-most lane with their kDa noted. Figure S2. Protein analysis of ZEB1, uPAR and Vimentin in MDA-MB-231 cells. Western Blots for the proteins shown in Figure 4. MDA-MB-231 cells were treated with DMSO or compound TAK-733 at the concentrations indicated or additional 4 Gy irradiation for 48 h or 72 h. Full membranes were separated at 75 kDa and 35 kDa and blots were probed with antibody for the protein indicated on the left. Molecular weight markers are shown in the right-most lane with their kDa noted. Data not included in the manuscript are crossed out. Figure S3. Migration activity of SKBR3 cells after irradiation. (a) Exemplary wound healing of SKBR3-GFP control (EV) cells after 0 and 24 h (scale bar: 25 µm) upon 0, 2 Gy, 4 Gy and 8 Gy irradiation, (b) Quantification of the wound healing capacity with the Image Colour Analyser determines the number of green pixels within the gap after 24 h and irradiation doses indicated [*n* = 3; ±SD; (*t*-test) * *p*-value < 0.05]. (c) Combined relative cell viability and caspase 3/7 luciferase activity assay normalized to non-irradiated SKBR3 cells 24 h after 0, 2, 4 and 8 Gy irradiation, [*n* = 3; ±SD; (*t*-test) *p*-value: ** < 0.01].

## References

[1] Darby S., McGale P., Correa C., Taylor C., Arriagada R., Clarke M., Cutter D., Davies C., Ewertz M., Early Breast Cancer Trialists’ Collaborative Group (2011). Effect of radiotherapy after breast-conserving surgery on 10-year recurrence and 15-year breast cancer death: Meta-analysis of individual patient data for 10,801 women in 17 randomised trials. Lancet.

[2] Alabdulkareem H., Pinchinat T., Khan S., Landers A., Christos P., Simmons R., Moo T.A. (2018). The impact of molecular subtype on breast cancer recurrence in young women treated with contemporary adjuvant therapy. Breast J..

[3] Keklikoglou I., Cianciaruso C., Guc E., Squadrito M.L., Spring L.M., Tazzyman S., Lambein L., Poissonnier A., Ferraro G.B., Baer C. (2019). Chemotherapy elicits pro-metastatic extracellular vesicles in breast cancer models. Nat. Cell Biol..

[4] Xu L., Wu X., Hu C., Zhang Z., Zhang L., Liang S., Xu Y., Zhang F. (2016). A meta-analysis of combination therapy versus single-agent therapy in anthracycline- and taxane-pretreated metastatic breast cancer: Results from nine randomized Phase III trials. Oncotargets Ther..

[5] Miles D., von Minckwitz G., Seidman A.D. (2002). Combination versus sequential single-agent therapy in metastatic breast cancer. Oncologist.

[6] Dhillon A.S., Hagan S., Rath O., Kolch W. (2007). MAP kinase signalling pathways in cancer. Oncogene.

[7] Roberts P.J., Der C.J. (2007). Targeting the Raf-MEK-ERK mitogen-activated protein kinase cascade for the treatment of cancer. Oncogene.

[8] Olea-Flores M., Zuniga-Eulogio M.D., Mendoza-Catalan M.A., Rodriguez-Ruiz H.A., Castaneda-Saucedo E., Ortuno-Pineda C., Padilla-Benavides T., Navarro-Tito N. (2019). Extracellular-Signal Regulated Kinase: A Central Molecule Driving Epithelial-Mesenchymal Transition in Cancer. Int. J. Mol. Sci..

[9] Song J., Ouyang Y., Che J., Li X., Zhao Y., Yang K., Zhao X., Chen Y., Fan C., Yuan W. (2017). Potential Value of miR-221/222 as Diagnostic, Prognostic, and Therapeutic Biomarkers for Diseases. Front. Immunol..

[10] Falkenberg N., Anastasov N., Rappl K., Braselmann H., Auer G., Walch A., Huber M., Hofig I., Schmitt M., Hofler H. (2013). MiR-221/-222 differentiate prognostic groups in advanced breast cancers and influence cell invasion. Br. J. Cancer.

[11] Liang Y.K., Lin H.Y., Dou X.W., Chen M., Wei X.L., Zhang Y.Q., Wu Y., Chen C.F., Bai J.W., Xiao Y.S. (2018). MiR-221/222 promote epithelial-mesenchymal transition by targeting Notch3 in breast cancer cell lines. NPJ Breast Cancer.

[12] Stinson S., Lackner M.R., Adai A.T., Yu N., Kim H.J., O’Brien C., Spoerke J., Jhunjhunwala S., Boyd Z., Januario T. (2011). TRPS1 targeting by miR-221/222 promotes the epithelial-to-mesenchymal transition in breast cancer. Sci. Signal..

[13] Shah M.Y., Calin G.A. (2011). MicroRNAs miR-221 and miR-222: A new level of regulation in aggressive breast cancer. Genome Med..

[14] Zhao J.J., Lin J., Yang H., Kong W., He L., Ma X., Coppola D., Cheng J.Q. (2008). MicroRNA-221/222 negatively regulates estrogen receptor alpha and is associated with tamoxifen resistance in breast cancer. J. Biol. Chem..

[15] Miller T.E., Ghoshal K., Ramaswamy B., Roy S., Datta J., Shapiro C.L., Jacob S., Majumder S. (2008). MicroRNA-221/222 confers tamoxifen resistance in breast cancer by targeting p27Kip1. J. Biol. Chem..

[16] Rao X., Di Leva G., Li M., Fang F., Devlin C., Hartman-Frey C., Burow M.E., Ivan M., Croce C.M., Nephew K.P. (2011). MicroRNA-221/222 confers breast cancer fulvestrant resistance by regulating multiple signaling pathways. Oncogene.

[17] Li W., Guo F., Wang P., Hong S., Zhang C. (2014). miR-221/222 confers radioresistance in glioblastoma cells through activating Akt independent of PTEN status. Curr. Mol. Med..

[18] Tang Z., Li C., Kang B., Gao G., Li C., Zhang Z. (2017). GEPIA: A web server for cancer and normal gene expression profiling and interactive analyses. Nucleic Acids Res..

[19] Anastasov N., Hofig I., Radulovic V., Strobel S., Salomon M., Lichtenberg J., Rothenaigner I., Hadian K., Kelm J.M., Thirion C. (2015). A 3D-microtissue-based phenotypic screening of radiation resistant tumor cells with synchronized chemotherapeutic treatment. BMC Cancer.

[20] Falkenberg N., Hofig I., Rosemann M., Szumielewski J., Richter S., Schorpp K., Hadian K., Aubele M., Atkinson M.J., Anastasov N. (2016). Three-dimensional microtissues essentially contribute to preclinical validations of therapeutic targets in breast cancer. Cancer Med..

[21] Mahapatra D.K., Asati V., Bharti S.K. (2017). MEK inhibitors in oncology: A patent review (2015-Present). Expert Opin. Ther. Pat..

[22] Peng D.H., Kundu S.T., Fradette J.J., Diao L., Tong P., Byers L.A., Wang J., Canales J.R., Villalobos P.A., Mino B. (2019). ZEB1 suppression sensitizes KRAS mutant cancers to MEK inhibition by an IL17RD-dependent mechanism. Sci. Transl. Med..

[23] Dong Q., Dougan D.R., Gong X., Halkowycz P., Jin B., Kanouni T., O’Connell S.M., Scorah N., Shi L., Wallace M.B. (2011). Discovery of TAK-733, a potent and selective MEK allosteric site inhibitor for the treatment of cancer. Bioorg. Med. Chem. Lett..

[24] Santarpia L., Lippman S.M., El-Naggar A.K. (2012). Targeting the MAPK-RAS-RAF signaling pathway in cancer therapy. Expert Opin Ther. Targets.

[25] Lee S., Rauch J., Kolch W. (2020). Targeting MAPK Signaling in Cancer: Mechanisms of Drug Resistance and Sensitivity. Int. J. Mol. Sci..

[26] Karoulia Z., Gavathiotis E., Poulikakos P.I. (2017). New perspectives for targeting RAF kinase in human cancer. Nat. Rev. Cancer.

[27] Lito P., Rosen N., Solit D.B. (2013). Tumor adaptation and resistance to RAF inhibitors. Nat. Med..

[28] Nebbioso A., Tambaro F.P., Dell’Aversana C., Altucci L. (2018). Cancer epigenetics: Moving forward. PLoS Genet.

[29] Fardi M., Solali S., Farshdousti Hagh M. (2018). Epigenetic mechanisms as a new approach in cancer treatment: An updated review. Genes Dis..

[30] Bennett R.L., Licht J.D. (2018). Targeting Epigenetics in Cancer. Annu. Rev. Pharm. Toxicol..

[31] Li S., Li Q., Lu J., Zhao Q., Li D., Shen L., Wang Z., Liu J., Xie D., Cho W.C. (2019). Targeted Inhibition of miR-221/222 Promotes Cell Sensitivity to Cisplatin in Triple-Negative Breast Cancer MDA-MB-231 Cells. Front. Genet..

[32] Ruggeri C., Gioffre S., Achilli F., Colombo G.I., D’Alessandra Y. (2018). Role of microRNAs in doxorubicin-induced cardiotoxicity: An overview of preclinical models and cancer patients. Heart Fail. Rev..

[33] Kakimoto Y., Tanaka M., Hayashi H., Yokoyama K., Osawa M. (2018). Overexpression of miR-221 in sudden death with cardiac hypertrophy patients. Heliyon.

[34] McCubrey J.A., Steelman L.S., Chappell W.H., Abrams S.L., Wong E.W., Chang F., Lehmann B., Terrian D.M., Milella M., Tafuri A. (2007). Roles of the Raf/MEK/ERK pathway in cell growth, malignant transformation and drug resistance. Biochim. Biophys. Acta.

[35] Nitsche M., Pahl R., Huber K., Eilf K., Dunst J. (2015). Cardiac Toxicity after Radiotherapy for Breast Cancer: Myths and Facts. Breast Care (Basel).

[36] Hanzelmann S., Castelo R., Guinney J. (2013). GSVA: Gene set variation analysis for microarray and RNA-seq data. BMC Bioinform..

[37] Anastasov N., Klier M., Koch I., Angermeier D., Hofler H., Fend F., Quintanilla-Martinez L. (2009). Efficient shRNA delivery into B and T lymphoma cells using lentiviral vector-mediated transfer. J. Hematop..

[38] Anastasov N., Hofig I., Vasconcellos I.G., Rappl K., Braselmann H., Ludyga N., Auer G., Aubele M., Atkinson M.J. (2012). Radiation resistance due to high expression of miR-21 and G2/M checkpoint arrest in breast cancer cells. Radiat. Oncol..

[39] Anastasov N., Hofig I., Mall S., Krackhardt A.M., Thirion C. (2016). Optimized Lentiviral Transduction Protocols by Use of a Poloxamer Enhancer, Spinoculation, and scFv-Antibody Fusions to VSV-G. Methods Mol. Biol..

[40] Anastasov N., Bonzheim I., Rudelius M., Klier M., Dau T., Angermeier D., Duyster J., Pittaluga S., Fend F., Raffeld M. (2010). C/EBPbeta expression in ALK-positive anaplastic large cell lymphomas is required for cell proliferation and is induced by the STAT3 signaling pathway. Haematologica.

[41] Hofig I., Atkinson M.J., Mall S., Krackhardt A.M., Thirion C., Anastasov N. (2012). Poloxamer synperonic F108 improves cellular transduction with lentiviral vectors. J. Gene Med..

[42] Falkenberg N., Anastasov N., Schaub A., Radulovic V., Schmitt M., Magdolen V., Aubele M. (2015). Secreted uPAR isoform 2 (uPAR7b) is a novel direct target of miR-221. Oncotarget.

[43] Huber M.C., Mall R., Braselmann H., Feuchtinger A., Molatore S., Lindner K., Walch A., Gross E., Schmitt M., Falkenberg N. (2016). uPAR enhances malignant potential of triple-negative breast cancer by directly interacting with uPA and IGF1R. BMC Cancer.

[44] Aubele M., Auer G., Walch A.K., Munro A., Atkinson M.J., Braselmann H., Fornander T., Bartlett J.M. (2007). PTK (protein tyrosine kinase)-6 and HER2 and 4, but not HER1 and 3 predict long-term survival in breast carcinomas. Br. J. Cancer.

[45] Mutschelknaus L., Azimzadeh O., Heider T., Winkler K., Vetter M., Kell R., Tapio S., Merl-Pham J., Huber S.M., Edalat L. (2017). Radiation alters the cargo of exosomes released from squamous head and neck cancer cells to promote migration of recipient cells. Sci. Rep..

