# Supplementary figures and images for: MEK1 Inhibitor Combined with Irradiation Reduces Migration of Breast Cancer Cells Including miR-221 and ZEB1 EMT Marker Expression
Source: Cancers (Basel). 2020 Dec 14;12(12):3760. doi: 10.3390/cancers12123760 (PMC7764972; doi:10.3390/cancers12123760)

**Figure S1**

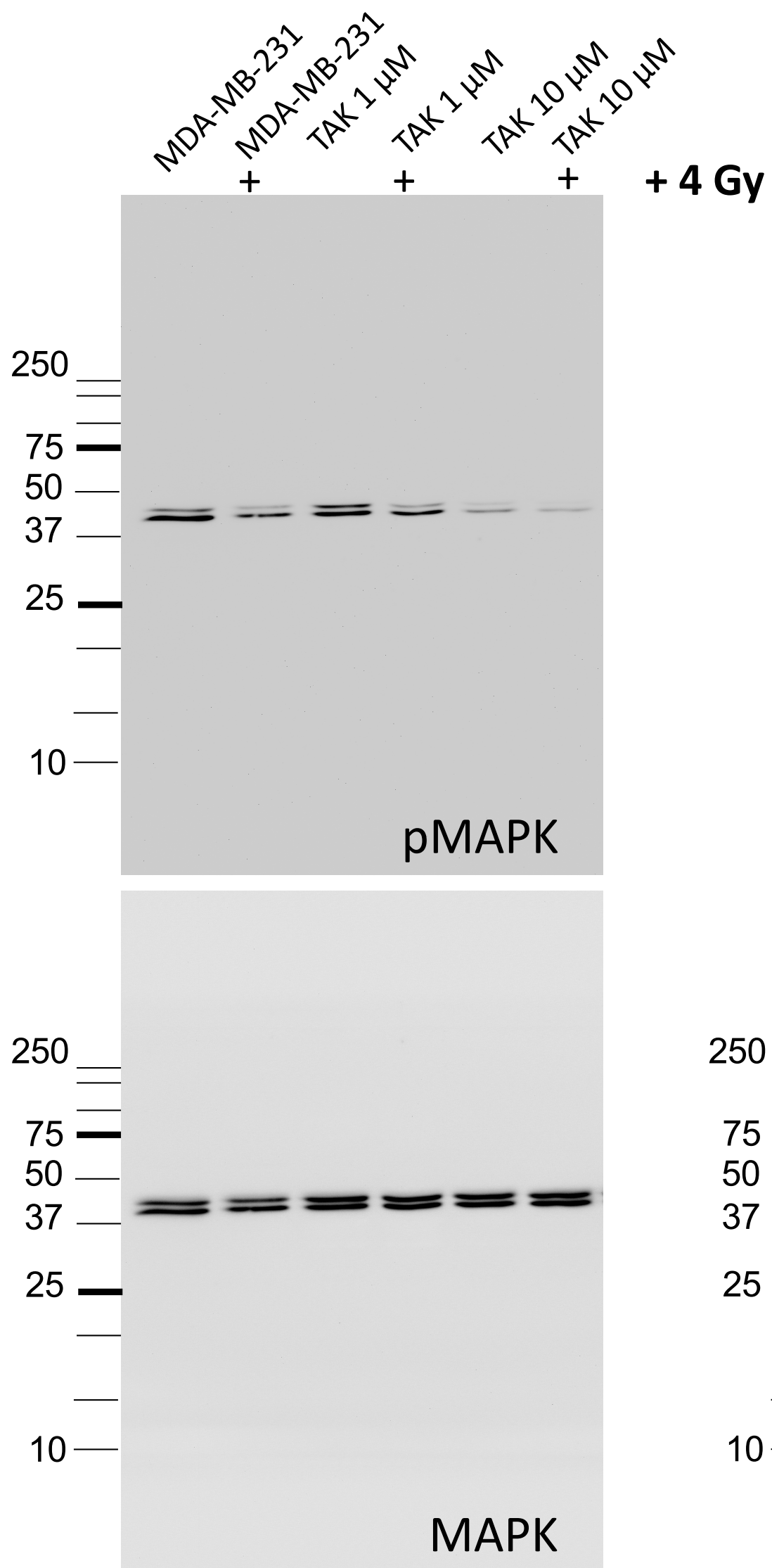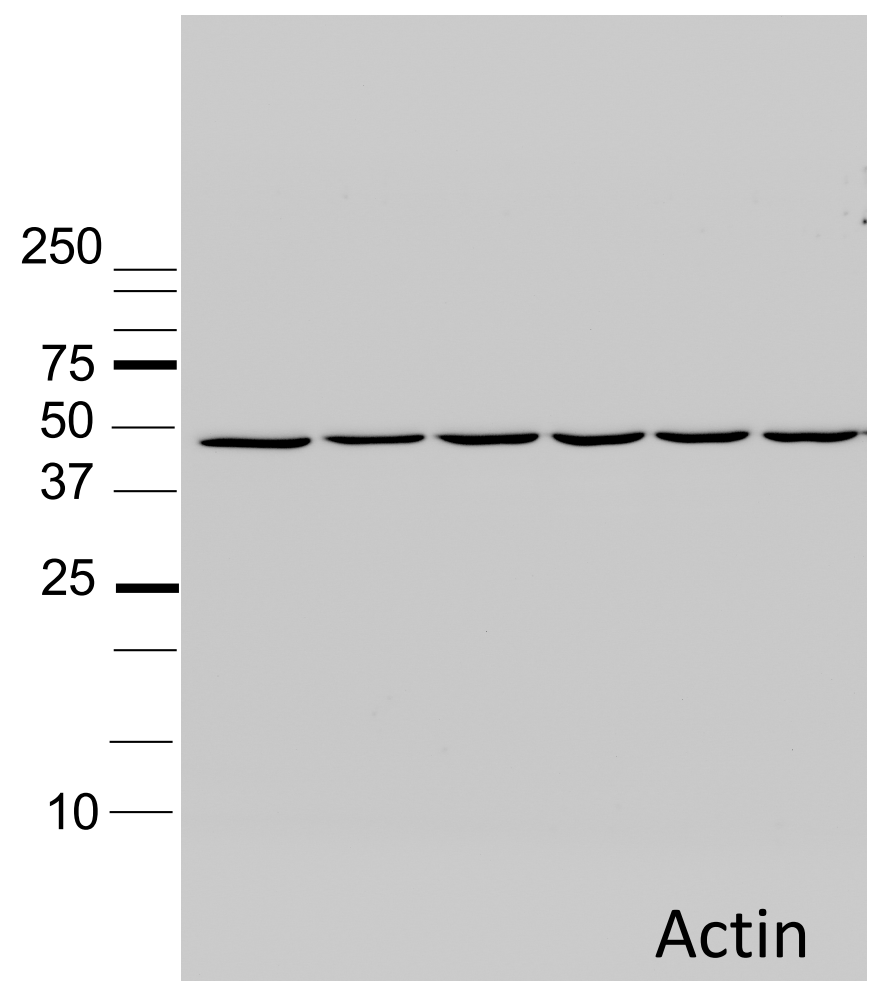

Supplement: Supplementary file 1 [file cancers-12-03760-s001.zip › FigureS1.pdf]

Figure S2

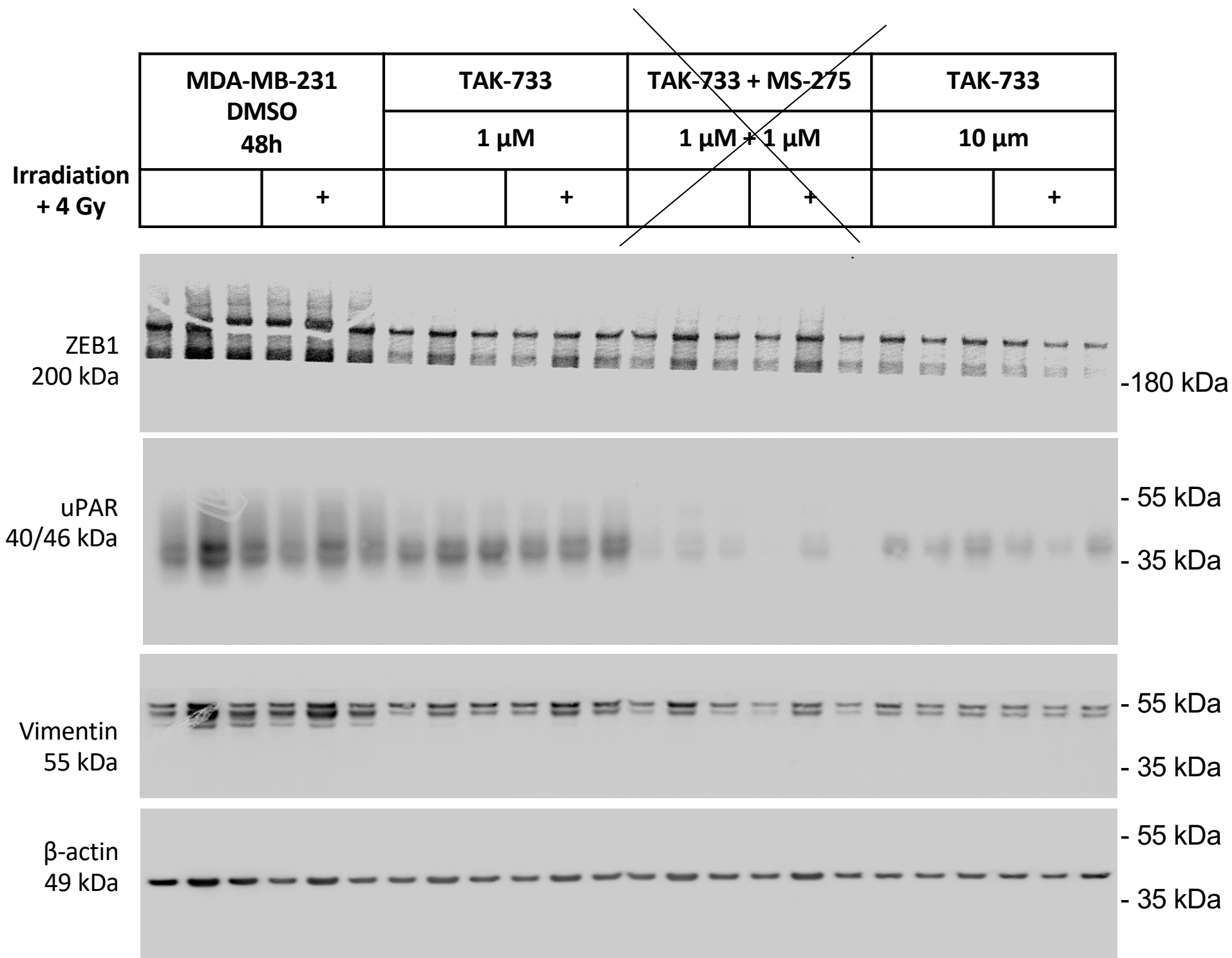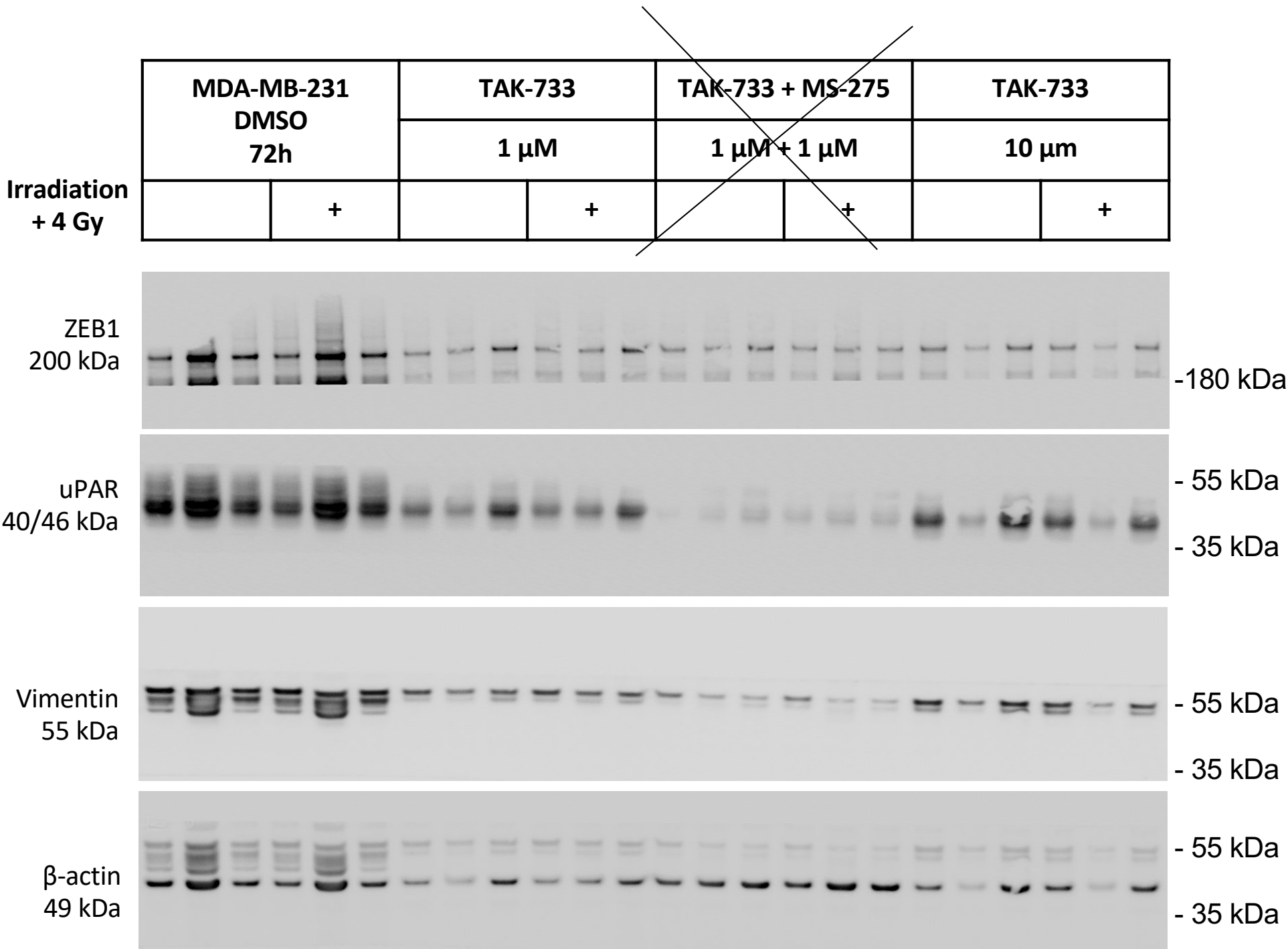

Supplement: Supplementary file 1 [file cancers-12-03760-s001.zip › FigureS2.pdf]

Figure S3

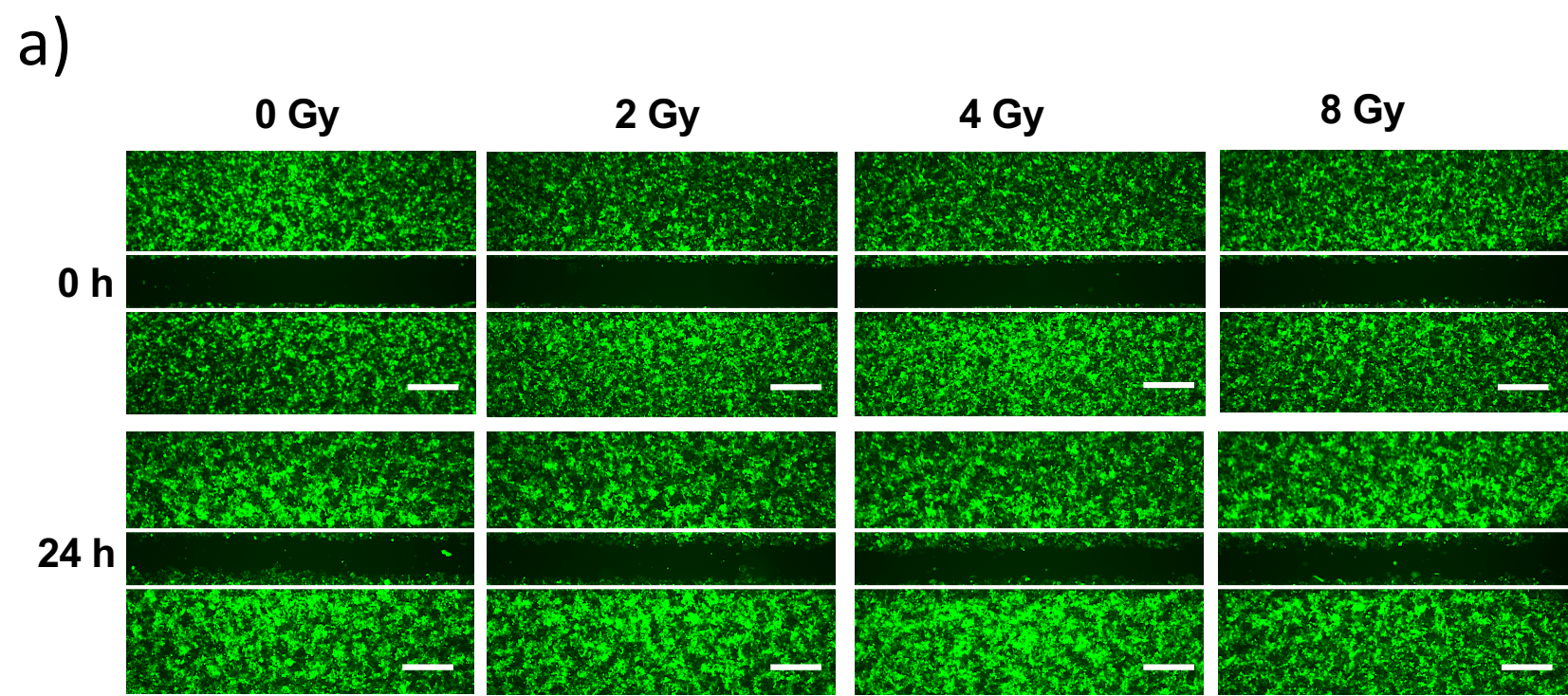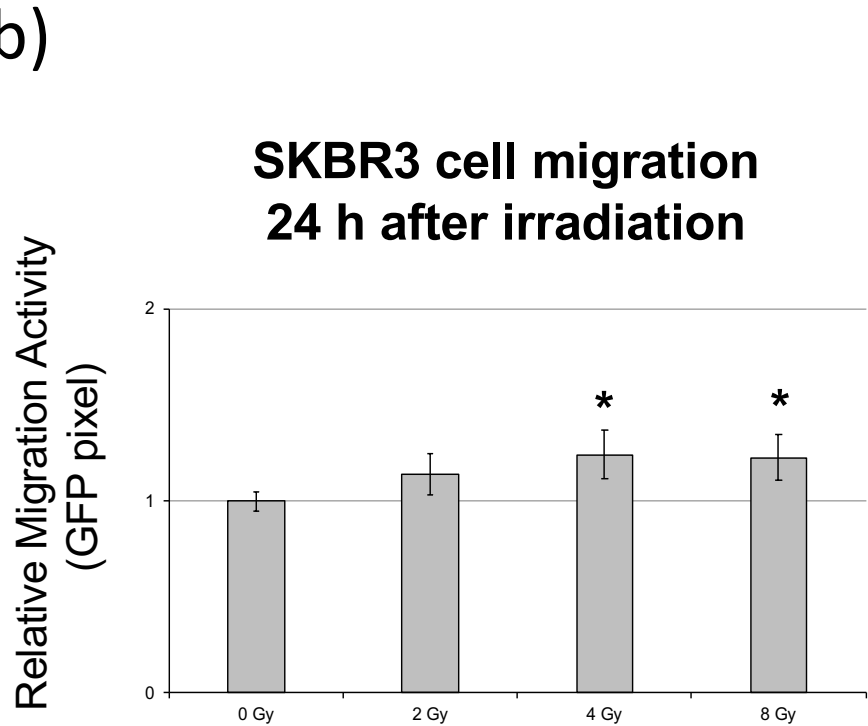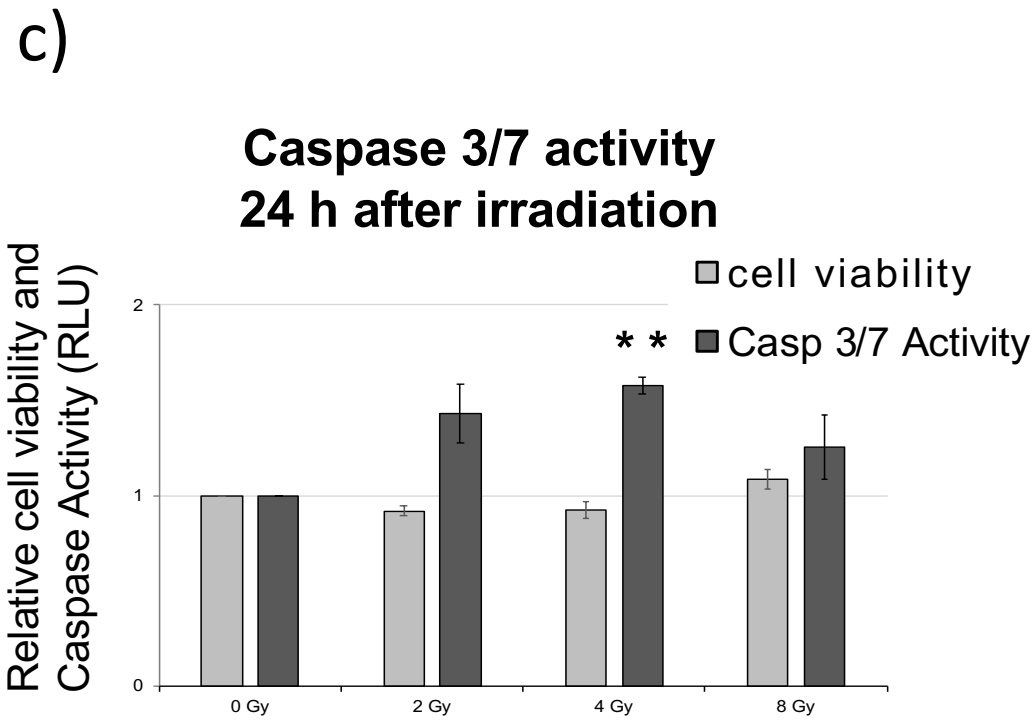

Supplement: Supplementary file 1 [file cancers-12-03760-s001.zip › FigureS3.pdf]
